# A Data Element-Function Conceptual Model for Data Quality Checks

**DOI:** 10.5334/egems.289

**Published:** 2019-04-23

**Authors:** James R. Rogers, Tiffany J. Callahan, Tian Kang, Alan Bauck, Ritu Khare, Jeffrey S. Brown, Michael G. Kahn, Chunhua Weng

**Affiliations:** 1 Department of Biomedical Informatics, Columbia University, US; 2 University of Colorado Denver Anschutz Medical Campus, US; 3 Kaiser Permanente Northwest Center for Health Research, US; 4 Children’s Hospital of Philadelphia, US; 5 Department of Population Medicine, Harvard Medical School and Harvard Pilgrim Health Care Institute, US

**Keywords:** data quality, knowledge acquisition, clinical data research networks, electronic healthcare records, natural language processing

## Abstract

**Introduction:**

In aggregate, existing data quality (DQ) checks are currently represented in heterogeneous formats, making it difficult to compare, categorize, and index checks. This study contributes a data element-function conceptual model to facilitate the categorization and indexing of DQ checks and explores the feasibility of leveraging natural language processing (NLP) for scalable acquisition of knowledge of common data elements and functions from DQ checks narratives.

**Methods:**

The model defines a “data element”, the primary focus of the check, and a “function”, the qualitative or quantitative measure over a data element. We applied NLP techniques to extract both from 172 checks for Observational Health Data Sciences and Informatics (OHDSI) and 3,434 checks for Kaiser Permanente’s Center for Effectiveness and Safety Research (CESR).

**Results:**

The model was able to classify all checks. A total of 751 unique data elements and 24 unique functions were extracted. The top five frequent data element-function pairings for OHDSI were Person-Count (55 checks), Insurance-Distribution (17), Medication-Count (16), Condition-Count (14), and Observations-Count (13); for CESR, they were Medication-Variable Type (175), Medication-Missing (172), Medication-Existence (152), Medication-Count (127), and Socioeconomic Factors-Variable Type (114).

**Conclusions:**

This study shows the efficacy of the data element-function conceptual model for classifying DQ checks, demonstrates early promise of NLP-assisted knowledge acquisition, and reveals the great heterogeneity in the focus in DQ checks, confirming variation in intrinsic checks and use-case specific “fitness-for-use” checks.

## Introduction

Widespread collection of clinical data in a computerized format, such as electronic health records (EHRs) and administrative claims, has made available an unprecedented amount of health care data for computational reuse [1,2]. These data promise to facilitate comparative effectiveness research, safety surveillance, and pragmatic trials, to name a few [3,4,5,6,7,8]. Large clinical data research networks–here within referred to as networks–have been established to capitalize on these research opportunities [9]. These networks employ different data architectures to support a variety of data uses [9,10]. For example, Kaiser Permanente’s Center for Effectiveness and Safety Research (CESR) is a clinical research network that utilizes its eight regional research centers for improving the health and well-being of its members and the general public [11,12]. PEDSnet is a learning health system that focuses on EHR data for pediatric related research [13]. Sentinel is an active surveillance program for medication safety using data from primarily claims-based data partners [14,15]. The National Patient-Center Clinical Research Network (PCORnet®) is a network of networks that consists of a large, highly representative set of patients for health research [16,17]. The Observational Health Data Sciences and Informatics (OHDSI) initiative was created in response to the differences in data models used by clinical data research networks in order to enable large scale analytics [18].

Poor data quality (DQ) is a potential threat to the discoveries from these data [19,20,21]. DQ is a multi-dimensional concept that is described by different terms [22,23,24,25,26,27]. To address the heterogeneity of the terminology, Kahn et al. created a harmonized DQ assessment terminology framework in order to provide a unified language [23]. In brief, the framework has three primary categories with corresponding subcategories: (1) Conformance, which refers to the data’s compliance to structural constraints, subcategorized as Value, Relational, or Calculation; (2) Completeness, which refers to the data’s presence in a particular context, subcategorized as Atemporal or Temporal; and (3) Plausibility, which refers to the data’s feasibility, subcategorized as Atemporal, Temporal, or Uniqueness. Of note, Atemporal refers to a single instance in time while Temporal refers to multiple instances across a specified time period. Overlaid on these categories and subcategories are two assessment categories that further detail how to check expectations that a particular quality metric has been achieved: (1) verification, which focuses on internal expectations and (2) validation, which focuses on external expectations.

Multiple networks have established customized DQ checks corresponding to this framework, where current existing DQ checks tend to be empirically defined for network-specific purposes [28,29,30,31,32]. We see an unexplored opportunity to learn from these checks for harmonizing DQ checking methods, understanding their similarities and differences, sharing best practices, and moving towards community-based standardization of DQ checking efforts. A conceptual model for DQ check content promises to better enable aggregate analyses of DQ checks.

The primary goal of this study is to propose a conceptual model for indexing and categorizing DQ checks. The secondary goal of this study is to assess the feasibility of using natural language processing (NLP) methods for scalable acquisition of knowledge from narrative DQ checks accompanying the model, similar to other work [33]. This study contributes a foundational data element-function conceptual model and knowledge of data elements and functions in the DQ checks from two example networks.

## Methods

**Figure 1:**
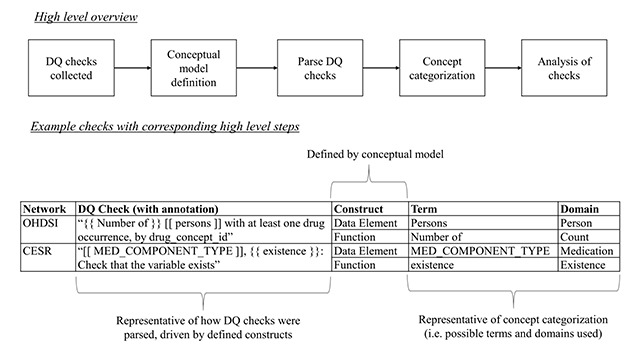
High-level overview of workflow with example DQ checks and their corresponding constructs, terms, and suggested domains. A data element is a focus of a DQ check (annotation is represented by “[[ *data element* ]]” for parsing); a function is the qualitative or quantitative evaluation over the data element (annotation is represented by “{{ *function* }}” for parsing). Each DQ check is essentially a function of a data element.

Figure 1 presents a high-level overview of this study. A set of DQ checks was collected from multiple networks. A subset of checks was reviewed to define a potential conceptual model for categorization of the DQ checks. We used the proposed conceptual model as a guide to annotate and parse all DQ checks. Because of the wide variety of terms extracted, we categorized terms into broader categories for easier interpretation. We concluded the study with a descriptive analysis of our findings. Each subsequent subsection provides further details for each step. The IRB determined this study to be exempt.

### DQ checks collected

We evaluated DQ checks of two notable networks: OHDSI and CESR. We utilized previously collected checks from a prior study, which provided 172 checks from OHDSI and 3,434 checks from CESR. Checks were stored as narrative text in Microsoft Office Excel 2016 spreadsheets [29]. Specific to OHDSI, we analyzed 67 additional DQ checks available from another study and stored them similarly, leading to a total of 239 checks from OHDSI [34]. In brief, the difference between the 172 OHDSI checks and the 67 additional OHDSI checks is that the former are pre-computational while the latter are both derived and community added [31,34].

### Conceptual model definition

The overall guiding principle of the conceptual model was to establish the most fundamental constructs needed to represent and index DQ checks in a common format. In order to define potential constructs, a random 10 percent sample of DQ checks were reviewed from each network. Following the aforementioned principle, the review led to the proposal of two constructs: 1) a “data element”, which is the primary focus of the DQ check, and 2) a “function”, which is the qualitative or quantitative measure applied to the data element; Figure 1 provides examples of these constructs. We expect that all DQ checks have these two constructs. We refer to this conceptual model as the data element-function model.

This setup was chosen as it echoes the entity-attribute-value (EAV) approach utilized in modeling heterogeneous data [35]. EAV was chosen because of its simplicity, common use, and flexibility for extension. In brief, an entity is a particular object of interest; an attribute is a descriptive of the entity; and the value is the quantity of the attribute. For this study, the entity is analogous to the individual DQ check; the possible attributes are the constructs (either data elements or functions); and the values are to be extracted from the collected DQ checks.

### Parse DQ checks

Using the conceptual model as a guide, we extracted terms corresponding to data elements and functions using NLP models. To do so, a multi-step procedure was followed per network. The procedure was stratified by network because the DQ checks were in different narrative formats. The DQ checks were split into a training set and a testing set, with the aforementioned 10 percent sample serving as the training set while the remaining 90 percent of checks served as the testing set. We chose not to expand upon our 10 percent sample for training because the highly structured and consistent nature of the majority of DQ checks we reviewed did not reveal sophisticated linguistic patterns, making it feasible for the reuse of an existing NLP system as is with minimal training. Regarding annotation, we chose a subset from the training set to capture any of the observed changes in linguistic patterns. For CESR, 10 checks were chosen while for OHDSI, 5 checks were chosen.

After the subsets were selected, an iterative annotation process by authors JRR and CW was pursued. Annotations were driven by defined constructs of the model, with sample checks presented in Figure 1. For example, the DQ check “Number of persons with at least one drug occurrence, by drug_concept_id” has the data element “persons” and the function “number of”. We revisit the existence of the “by drug_concept_id” subsetting clause in the Discussion.

NLP models were defined and applied on the annotated training set of DQ checks. Models optimized for extracting data elements and functions from DQ checks were implemented in an open-source NLP system [36]. The NLP system chosen was originally intended for clinical trial eligibility criteria, but we repurposed portions of its pipeline, specifically the named entity recognition methodology, to be applied on textual DQ checks. Only one data element and only one function must be identified from each DQ check because each check is expected to have one intended focus. Annotation guidelines ensured only one data element and one function could be identified, with relevant terms extracted based on the structural context of each check. The model evaluation metric for each construct was the proportion of correctly identified constructs divided by the total number of possible constructs, which equates to the total number of DQ checks analyzed. A threshold of 90 percent for this metric was set for each construct with author JRR verifying whether or not the appropriate term was identified for each check. If the threshold was not met for either construct for the specific network, then additional checks were annotated based on the checks that were not appropriately identified by the NLP model and the NLP models were re-trained. This process was iterated until an acceptable performance was achieved.

After sufficient performance was achieved for both constructs, the NLP models were applied to the testing set. Proportions of correctly identified constructs per each network were evaluated, with author JRR manually reviewing each DQ check to determine whether or not the appropriate term for each construct was identified. If the incorrect term was identified, the reviewing author noted it and manually derived the appropriate construct that would be used for analysis. From there, the constructs were organized into domains. The NLP system was programed in Python 2.7.

### Concept categorization

Constructs derived from the NLP results were manually categorized into domains. For data elements, we leveraged the domain definitions in OHDSI because they are expected to be robust to many observational database schemas [37]. Where there was no relevant OHDSI domain, authors JRR and CW added one [38]. Example domains for data elements include conditions, medications, procedures, and social history. Domains for functions were related to concepts akin to how the data elements were aggregated. For example, “exist” and “existence” would both be represented by the domain “existence”. All domains were initially determined by author JRR, reviewed by author CW, and then adjudicated based on consensus. Figure 1 presents sample terms for each domain.

### Analysis of checks

Descriptive statistics for the categorized domains per network was evaluated. The categorized domains were also compared with labels from the DQ harmonization framework in order to provide a sense of how the domains are intrinsically applied; these labels were already derived from prior work [29]. In order to assess relations between data element domains and function domains, heat maps were created. We also compared overlaps in domains between OHDSI and CESR to assess robustness in domain categorization. Analyses were performed in R 3.4.2.

## Results

### Feasibility to leverage NLP to scale knowledge acquisition for standardizing DQ checks

There was a total of 239 DQ checks for OHDSI. The additional 67 checks were in a narrative format not conducive for NLP processing, so only the 172 DQ checks were parsed by NLP and included in the main analysis (results of manual curation for the 67 checks are presented in the Supplemental Material). There were 18 DQ checks used in the training phase, with 100 percent of the data elements and functions from the training set correctly identified. For the remaining 154 DQ checks from the testing set, 76 percent and 98 percent of data elements and functions were correctly identified, respectively. There were 3,434 DQ checks for CESR parsed by NLP. For the 344 DQ checks in the training phase, 97 percent and 92 percent of data elements and functions were correctly identified, respectively, after one iteration of annotation. For the remaining 3,090 DQ checks in the testing phase, 97 percent and 89 percent of data elements and functions were correctly identified, respectively.

### Concept categorization – suggested domains and allowable syntax

Table 1 presents 49 unique data elements mapped to 12 domains extracted from the 172 OHDSI DQ checks. The domain with the most unique terms was Insurance (13 terms), followed by Medication (10), and then Condition, Observation, and Visit (5 each). In regards to frequency, the most common domains for data elements in the OHDSI DQ checks were Person (55; 32 percent), followed by Insurance (23; 13 percent), Medication (20; 12 percent), Observations (16; 9 percent), and Condition (15; 9 percent). For functions, there were a total of 3 unique domains: Count, Distribution, and Time Length. The most common domain for functions was Count (128; 74 percent). In general, the functions reflected assessments related to summary-level evaluations, such as providing a count of a data element relative to a particular specification.

**Table 1:** Allowable terms for domain descriptions of OHDSI DQ check constructs.

Domain	Domain Definition	Terms	Count of Unique Terms in Domain	Count of Checks in Domain

*Data Elements (DEs)*				

Age	DE related to age-specific variables	age at first observation period; age at death; age	3	10
Care Site	DE related to places of care variables	care sites	1	3
Condition	DE related to condition-specific variables	condition occurrence records; condition occurrence concepts; condition eras; condition era length; condition era concepts	5	15
Death	DE related to death-specific variables	death records; records of death; time from death	3	9
Insurance	DE related to insurance-specific variables	procedure cost records; total paid; total out-of-pocket; paid toward deductible; paid copay; paid coinsurance; paid by payer; paid by coordination of benefit; ingredient_cost; drug cost records; dispensing fee; average wholesale price; payer plan (days) of first payer plan period	13	23
Medication	DE related to medication-specific variables	refills; quantity; drug occurrence records; drug exposure records; drug exposure concepts; drug eras; drug era records; drug era length; drug era concepts; days_supply	10	20
Numeric Values	DE related to an unspecified numeric values	numeric values	1	1
Observations	DE related to observation-centric variables	records; observation records; observation occurrence records; observation occurrence concepts; observation (days) of first observation period	5	16
Person	DE that examines only persons	Persons	1	55
Procedure	DE related to procedure-specific variables	procedure occurrence records	1	8
Provider	DE related to provider-specific variables	Providers	1	3
Visit	DE related to visit-record related variables	visits; visit records; visit occurrence records; visit occurrence concepts; length of stay	5	9
*Functions*				

Count	Measures the count of DE relative to a certain specification (e.g., number of persons with X)	Number of	1	128
Distribution	Measures the dispersion of DE across a certain specification (e.g., distribution of age by X)	Distribution of	1	39
Time Length	Measures the time frame of DE given a certain specification (e.g., length of observation period (days) of first observation period by X)	Length of	1	5

**Figure 2:**
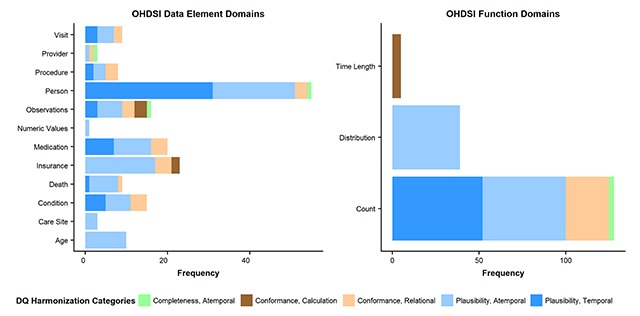
Horizontal bar charts of frequency of DQ check domains specific to OHDSI, overlaid with DQ harmonization categories. DQ Harmonization Categories brief descriptions: Completeness, Atemporal is the data’s presence in a particular context at an individual time point; Conformance, Calculation is the data’s compliance to constraints relating to computationally derived values from existing data; Conformance, Relational is the data’s compliance to structural constraints as it relates to physical database structure specifications (e.g., primary key and foreign key relationships); Plausibility, Atemporal is the data’s feasibility at an individual time point; Plausibility, Temporal is the data’s feasibility across a series of time points in a defined time period.

Figure 2 presents bar charts of the frequencies of the DQ harmonization categories for all DQ check domains specific to OHDSI. The majority of data element domains focused on Atemporal Plausibility followed by Temporal Plausibility. In terms of function domains, DQ checks that measured Counts tended to focus on either Atemporal Plausibility or Temporal Plausibility whereas DQ checks that measured Distributions focused only on Atemporal Plausibility checks and DQ checks that measured Time Length only focused on Calculation Conformance checks. Visual mapping of the OHDSI DQ check domains to DQ harmonized framework categories in terms of percentages are also available (Supplemental Material, Figure S1).

Table 2 presents the frequent data elements extracted from the 3,434 CESR DQ checks. There was a total of 702 unique data elements categorized into 23 domains. The domain with the most unique data elements was Medication (120 terms), followed by Socioeconomic Factors (117), Tumor (107), Social History (53), and Internal ID (53). The data elements were specific to an individual field in a data table, such as “birth date”, “code type”, or “smoking use”. In regards to frequency, the most common data element domains represented in the CESR DQ checks were Medication (764; 22 percent), Tumor (534; 16 percent), Socioeconomic Factors (464; 14 percent), Social History (216; 6 percent), and Visit (190; 6 percent). For functions, there were a total of 21 unique functions that were categorized into 14 domains. Four domains, i.e., Consistency, Count, Existence, and Overlap, contained more than one function. The domains with the most common functions were Variable Type (726; 21 percent), Missing (722; 21 percent), Existence (715; 21 percent), Variable Length (467; 14 percent), and Count (380; 11 percent). In general, most of the functions reflected variable-level assessments.

**Table 2:** Allowable terms for domain descriptions of CESR DQ check constructs.

Domain	Domain Definition	Sample Term Descriptions*	Count of Unique Terms in Domain**	Count of Checks in Domain

*Data Elements (DEs)*				

Birth	DEs related to birth-related variables	birth date, bdate	4	11
Bone Measurement	DEs related to bone measurement variables	bone measured, machine type used, scan date	10	42
Care Site	DEs related to place of care specific variables	facility name	2	20
Condition	DEs related primarily to condition-specific variables	principal diagnosis, diagnosis code type, original diagnosis	17	85
Date	DEs related to unspecified date variables	Date	2	24
Death	DEs related to death-specific variables	death date, age at death	6	24
Enrollment	DEs related to enrollment-specific variables	enrollment start date, enrollment end date, enrollment basis	11	30
Ethnicity	DEs related to ethnicity-specific variables	Hispanic	2	10
Gender	DEs related to gender-specific variables	gender	2	10
Internal ID	DEs defined by internally utilized constructions	protocol ID, row ID, template ID	53	144
Lab	DEs related to lab-specific variables	test type, specimen source, modification measures (e.g., high, low, etc.)	26	121
Language	DEs related to speaking language variables	primary language, need for interpreter, language usage	8	36
Medication	DEs related to medication-specific variables	refills, quantity, dosage form, dosage amount, order date, prescription date, infusion duration	120	764
MRN	DEs related to medical record numbers	general MRNs, table-specific MRNs (such as related to enrollment)	12	153
Observations	DEs related to ambiguous variables	unit of measure, type of activity, message-related characteristics	43	184
Procedure	DEs related to procedure-specific variables	CPT modifiers, procedure date, original procedure	8	43
Provider	DEs related to provider-specific variables	specialty, provider demographics, provider type	26	146
Race	DEs related to race-specific variables	race listed (i.e., “race1, race2, etc.”), race cross-section with ethnicity (e.g., non-Hispanic white)	24	97
Social History	DEs related to social history variables	smoking use, alcohol use, drug use	53	216
Socioeconomic Factors	DEs related to socioeconomic variables	household income, poverty status, education level, insurance-related	117	464
Tumor	DEs related to cancer-specific variables	SSF measures, stages of progression, dates of particular cancer-related therapies (e.g., chemotherapy)	107	534
Visit	DEs related to visit-specific variables	inpatient length of stay, discharge status, admission type	29	190
Vital	DEs related to vital-specific variables	weight measurements, blood pressure measurements, pulse measurements	20	86
*Functions*				

Category	Examines whether or not appropriate categories of a DE are correctly entered	Category	1	243
Consistency	Examines if a target DE follows an expected pattern with another DE	Expected order of values; Compare to; Extra check; Consistency	4	10
Count	Measures the count of a DE (either by the DE or categories of the DE)	Frequency; Counts; Number	3	380
Cross tab	Cross-section of a target DE with other DEs	Cross tab	1	19
Distribution	Examines context-specific dispersion of a DE	Distribution	1	1
Existence	Examines if the DE itself is present	Exist; Existence	2	715
Link	Examines if DE is linked correctly	Link	1	41
Missing	Examines if a DE’s entries are present	Missing	1	722
Overlap	Examines multiple locations of DE occurrence	Not overlap; Overlap	2	3
Sum	Measures the sum of DEs (typically used for proportions that must add to 1)	Sum	1	49
Trend	Examines time fluctuation of a DE	Trend	1	29
Uniqueness	Examines if DE duplicates are present	Uniqueness	1	29
Variable Length	Examines the variable length of a DE	Length	1	467
Variable Type	Examines how the DE is stored or defined (e.g., date format, integer, etc.)	Type	1	726

* Note that select example data element terms and descriptions are provided because some terms are proprietary and some data elements have many terms. ** Unique terms include case sensitive representations; for example, “race1” and “RACE1” are counted as unique.

**Figure 3:**
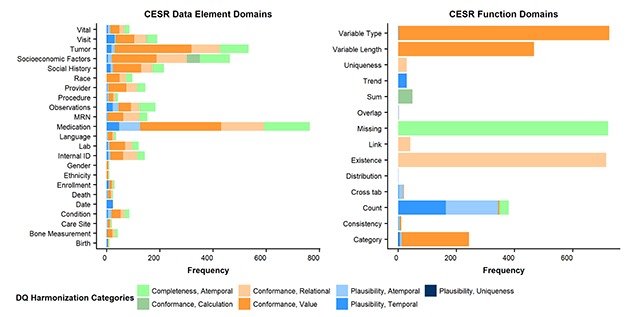
Horizontal bar charts of frequency of DQ check domains specific to CESR, overlaid with DQ harmonization categories. DQ Harmonization Categories brief descriptions: Completeness, Atemporal is the data’s presence in a particular context at an individual time point; Conformance, Calculation is the data’s compliance to constraints relating to computationally derived values from existing data; Conformance, Relational is the data’s compliance to structural constraints as it relates to physical database structure specifications (e.g., primary key and foreign key relationships); Conformance, Value is the data’s compliance to structural constraints as it relates to prespecified formatting constraints (e.g., data element is numeric); Plausibility, Atemporal is the data’s feasibility at an individual time point; Plausibility, Temporal is the data’s feasibility across a series of time points in a defined time period; Plausibility, Uniqueness is the data’s feasibility regarding duplication.

Figure 3 presents bar charts of the frequencies of the DQ harmonization categories for all DQ check domains specific to CESR. All data elements except for Date were part of checks that were focused on Value Conformance, Relational Conformance, and Atemporal Completeness. For functions, many domains pertained to one category of the DQ harmonization framework. Visual mapping of the CESR DQ check domains related to DQ harmonized framework categories in terms of percentages are also available (Supplemental Material, Figure S2).

### Knowledge acquisition – applicable pairs between data element domains and function domains

**Figure 4:**
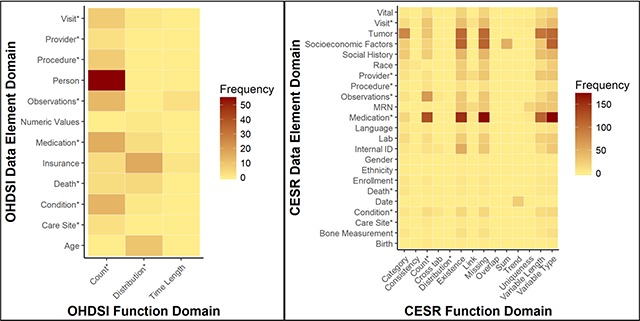
Heat maps of DQ check domains. Domains represented in both networks are indicated with an “*”.

Figure 4 presents heat maps of frequency for each pair of data element domain and function domain for both networks. Of the 172 DQ checks in OHDSI, the most common pair between data element domains and function domains was Person-Count (55; 32 percent of the DQ checks); in other words, there are 55 DQ checks examining the number of persons with a particular specification. The other most common pairs include Insurance-Distribution (17; 10 percent), Medication-Count (16; 9 percent), Condition-Count (14; 8 percent), and Observations-Count (13; 8 percent). In general, the majority of pairs found in OHDSI examine the frequency of a particular data element in a defined context.

Of the 3,434 DQ checks for CESR, almost all data element domains had DQ checks related to the five most common function domains. In particular, the most common pair was Medication-Variable Type (175; 5 percent of the data quality checks); in other words, there are 175 DQ checks that examine the variable type of medication-related data elements. The other most common pairs are Medication-Missing (172; 5 percent), Medication-Existence (152; 4 percent), Medication-Count (127; 4 percent), and Socioeconomic Factors-Variable Type (114; 3 percent). For some function domains, they were utilized mainly for one data element domain; specifically, all 49 of the Sum DQ checks were for Socioeconomic Factors, 24 of the 29 Trend DQ checks were for Date, and 18 of the 29 Uniqueness DQ checks were for MRN.

The heat map also illustrates overlapping domains between the two networks. There are 8 data element domains that overlap between them: Visit, Provider, Procedure, Observations, Medication, Death, Condition, and Care Site. Of those that do not overlap, there are some similarities that are associated but do not necessarily refer to same meaning and are thus not considered overlapping. For example, OHDSI has an Insurance domain that focuses primarily on insurance-related data elements; CESR has insurance-related data elements also, but those are represented by the broader domain Socioeconomic Factors. Similarly, OHDSI has an Age domain that focuses on the calculated value of age while CESR has a Birth domain that focuses on data elements that relate to the date of birth as opposed to the calculated age. As for functions, the overlapping domains are Count and Distribution. The description of length is utilized by both networks, but for different contexts: for OHDSI, length is in reference to a time frame whereas for CESR, length is in reference to a variable length.

## Discussion

### Key takeaways

The data element-function model provides a pragmatic setup for indexing and categorizing DQ checks. To populate this model with relevant knowledge, this study demonstrates promise that an NLP system can be used as a tool for large scale acquisition. For OHDSI, the reduction in the proportion of correctly identified data elements from the training set to the testing set was largely attributed to adjective modifiers used before the data element term that were not identified by our parser in the training phase. For example, if a check examined a data element with a distinct designation, the term “distinct” would be identified as opposed to the data element. For CESR, the slight drop in the proportion of correctly identified functions was attributed to checks with nuanced patterns not captured in the training set, in which the functions were found in a different portion of the check.

The similar format provided by the conceptual model enables comparisons between different networks’ DQ checks. When comparing the extracted terms from pre-computational OHDSI checks with the CESR checks, both organizations shared many similar focuses from a domain perspective. For example, both networks had checks regarding the counts of Medication, Observation, Provider, and Condition data elements.

However, differences in the content of the domains and how they were evaluated were prevalent between the two networks. One major difference is the granularity of data elements assessed. In OHDSI, the checks reflect aggregated data elements. This is expected as the purpose of these checks is to provide a way to examine the data at a summarized level and are applied after the data has been mapped to and extracted from the utilized common data model. For instance, many checks are based on aggregated record-level evaluations, such as drug occurrence records from the Medication domain, procedure cost records from the Procedure domain, and visit records from the Visit domain. In contrast, CESR checks were generally more granular and spanned across multiple components for the data elements, which in turn led to more terms represented. For example, CESR has checks that evaluate medications in the Medications domain, but the medication data was broken down into many aspects such as medication name, dosage form, and dosage unit. To emphasize the contrast between networks, one can compare data element domains found in similar function domains, such as counts in the Medication domain. OHDSI counts for Medication domain elements are on aggregated record-level evaluations, such as “drug exposure records” or “drug cost records”, whereas CESR focused on counts for individual components, such as number of medication names or number of dosage forms. This potentially reflects differing standards utilized by both networks’ underlying data models. For example, OHDSI utilizes the RxNorm terminology as a standard for all medications, where individual drug components can be categorized by higher level concepts, and lower-level concepts have the medication information built in (e.g., dosage) [18]. In contrast, CESR utilizes different medication terminologies (such as National Drug Codes), which have different representations that may lead to different kinds of checks [12].

This level of granularity also persists from the function perspective. Using the data element domain of Medication, OHDSI checks involve counts of particular medication-oriented data elements as well as the distribution of such data elements. However, CESR utilizes many checks from a variable perspective, such as whether the data element exists (Existence), if any values are missing (Missing), what is the stored length of the data element (Variable Length), what are the frequency of the values for the date element (Count), among other checks.

### Conceptual model in context of DQ heuristics

DQ checks are generally utilized for determining whether or not the data available is appropriate for particular tasks, also described as “fit-for-use” [39]. In the context of the data element-function model, the differences between the networks’ DQ checks provide evidence of differing purposes. For OHDSI, the checks represent a focus on specific “fitness-for-use” cases, as the checks provide a summary or characterization of data elements, which users must evaluate to determine if the data are acceptable for their goals after at least one extract-transfer-load (ETL) process. These particular checks are not intended to define an actionable strategy for correcting underlying errors, but are instead meant to flag potential issues for users to further evaluate. For the additional checks from OHDSI, they provide a more targeted focus of the data elements, but the functions on those data elements are still mostly reflective of “fitness-for-use” (such as thresholds and ratios). Ultimately, the OHDSI checks are a user-perspective endeavor, and it is up to the user to decide how to best address the findings of the checks. Of note, if users are interested in investigating different concerns prior to the aggregated checks, additional tools can be utilized [40].

For CESR, the checks tend to be at an intrinsic level because the focus is on ensuring certain data elements are present or accurately represented without reference to external requirements [23,27]. These checks are likely defined at this level because the DQ assessment is centralized to confirm the data meet basic expectations. As such, there is a central party responsible for making sure local contributing sites performed appropriate conversion of source data to the required common data model format before aggregated use. Put another way, the checks operate as a verification that the data are properly formatted and are ready for aggregated analyses. These observations are consistent with previous interpretations of these checks [29]. Of note, there exist other strategies that CESR utilizes beyond the collected CESR checks. These strategies are geared toward additional data characterization that is reviewed, compared to other local sites, and assessed for irregularities which require further investigation by local data teams. These strategies were not considered for this study.

Additionally, these results further extend the DQ harmonization framework by adding constructs and representations related to the predefined categories. In general, for both networks, data element domains tended to be categorized by many DQ harmonization categories whereas function domains were more homogenously captured. This provides a sense of face-validity: most data elements are checked in a variety of ways while the functions represent the kinds of checks utilized. For example, it is not surprising the CESR function domain Existence refers to Relational Conformance as the majority of those checks examined whether or not a particular variable existed; conversely, it is not surprising that virtually all CESR data element domains have Relational Conformance checks as each domain is likely evaluated to see whether or not particular variables exist. The different domains shed light on the kind of applications performed in context of the DQ harmonization framework.

### An alternative consideration for use cases beyond the conceptual model

The data element-function model defines foundational components for categorizing DQ checks, which fits its intended purpose to categorize DQ checks into their most basic components. As a result of this scope, caution must be exercised when considering whether or not the model is appropriate for certain use cases. Specifically, the model does not capture all information contained within DQ checks and the model is not set up to be executable. If these are of interest to a user, an alternative model to representing DQ checks is the Quality Data Model (QDM) with accompanying logic defined by clinical quality language (CQL) [41,42]. In brief, QDM defines clinical concepts in three segments: (1) a category to represent a general clinical concept; (2) a datatype to define a particular care process for a chosen category; and (3) an attribute to provide a specific detail of the overall concept. CQL provides the logic to perform evaluations on QDM elements and is designed to be “blind” to the underlying data structure.

To illustrate feasibility, a random 5 percent sample of DQ checks from each network were used for mapping into QDM-CQL syntax. Of the 9 OHDSI checks, only 5 (56 percent) were successfully mapped, while of the 172 CESR checks, only 44 (26 percent) were successfully mapped. For the OHDSI checks, the main reason for unmapped checks was because there was no appropriate QDM element to adequately define particular terms, specifically for insurance-related terms. For the CESR checks, the main reason for unmapped checks generally pertained to an inability to write the checks in a CQL format, specifically checks that focused on the underlying variable setup such as variable type or existence. This is expected as checks dependent on the underlying data model are beyond the scope of CQL.

Despite the limitation of not being able to map all sampled checks, the QDM-CQL model demonstrated advantageous characteristics. QDM-CQL was able to map multiple data elements (as opposed to primary data element of focus) within DQ checks. It was able to map attributes embedded within checks (such as “distinct” or temporal constrictions). It has already undergone significant development (i.e., syntax readily defined and can be executed). These advantages suggest the QDM-CQL model may work well for use cases focused on representing all relevant information from a DQ check as well as executing checks on particular data environments, conditional on the scope of the checks. Regardless, a more rigorous exploration is required.

### Limitations

This study serves as a proof of concept that constructs of DQ checks can be derived and categorized in a shared representation, but this model contains some limitations. First, the data element-function model has limited scope and focuses on a few aspects of DQ checks, which can lead to a loss of information of the DQ checks. For this study, we defined only two constructs that were expected to serve as a minimal definition of the DQ checks: what the primary focus is (i.e., data element) and how it is being evaluated (i.e., the function). Although this serves as a reasonable starting point for comparing DQ checks, it does not comprehensively capture all information. As per the sampled checks, the prevalence of under capturing information was higher for OHDSI checks than CESR checks. For the 18 sampled OHDSI checks, all of them had additional information, particularly related to stratifications. For example, using our procedure on the OHSDI DQ check “number of persons with at least one drug occurrence, by drug_concept_id” would not identify the information that this check is displaying a stratification by drug_concept_id and does not identify that each person is required to have at least one drug occurrence. For the 344 sampled CESR checks, 16 percent were identified as having additional information, with most prominent to related temporal considerations. For example, our procedure on the CESR DQ check “the count of encounter dates by year across all years of data” would not identify the temporal constraint of this check.

A second limitation is that the domain assignments are subjective, as certain constructs can be classified differently. For instance, the CESR data element of encounter dates could qualify as Visit and Date. For situations such as this, we would classify based on the most relevant category. For this example, we categorized it as Visit because the date was explicitly defined in relation to an encounter whereas the Date category referred to unspecified dates.

An additional consideration is that different domains could be constructed, depending on preference for broader or narrower categorization. For this study, we tried to focus on broad domains that were relatively informative, acknowledging that this is relatively subjective. For example, many of the domains defined in CESR could be specified as Demographics (such as Birth, Ethnicity, and Gender); we did not categorize by demographics because we agreed that these were broad enough to warrant their own domains. In contrast, a domain such as Medication contained many data elements (such as refills, quantity, and order date) but its data elements did not provide a broad enough scope to warrant their own categorizations. Similarly, some categories are vague because the data element that was identified was vague. For example, we had a category of Date for CESR–this is because the DQ checks looked at dates but did not provide specification of kind of dates (e.g., dates of birth, death, or discharge) within the checks themselves. These observations suggest a more rigorous categorical refinement should be considered. Despite this limitation, it is important to note that this was beyond the scope of this study as the overall goal was to prove that this step could be achieved and to provide a sense of plausibility for categorization.

A third limitation is that the NLP models are susceptible to overfitting. There was a substantial decrease in the proportion of correctly identified data elements in the test set versus the training set in the OHDSI dataset and, to a lesser extent, in the CESR results with a slight decrease in the proportion of correctly identified functions. In order to remedy this, the training and testing sets may require a more even split to capture more diverse checks for annotation.

A fourth limitation is the DQ checks collected do not necessarily represent all possible DQ checks that can be constructed. As mentioned earlier for both networks, other checking mechanisms exist but were not included in this study. Furthermore, the checks collected are focused on static datasets rather than comparing datasets between ETLs, which means the conceptual model does not include checks that examine dynamic changes.

### Future work

As a proof of concept, this study provides a multitude of directions for future work. One direction is to expand the scope of the data element-function model to consider extracting additional constructs from the defined DQ checks. As found above, the focus on just two constructs provided general descriptions of what checks exist but came at the expense of a loss in information. Refining constructs to be derived from the DQ checks could lead to a more thorough interpretation of the kinds of checks that are applied and further enhance the interpretation for fitness-for-use. In-depth models from other terminologies, such as Systematized Nomenclature of Medicine–Clinical Terms (SNOMED-CT), could serve as a guide to refine how additional constructs are defined and utilized [43]. In a similar vein, terminologies themselves may be utilized to more rigorously define appropriate domains for extracted terms.

A second direction is to test the robustness of the data element-function model with accompanying NLP procedure on additional networks. As observed in OHDSI and CESR, organizational differences persist and were echoed in the setup of their DQ checks. Future work could further evaluate whether or not this observation persists when examining other organizations that have readily available DQ checks, such as Sentinel, PEDSnet, and PCORNet [17,29]. However, this would be dependent on the DQ checks being stored as narrative text.

One last direction is to more formally compare the data element-function model to alternative models in order to examine which is optimal for particular use cases. As explored above, the model can work well for providing categorizations of DQ checks for comparisons while the QDM-CQL model has potential to work well for executable DQ checks. Particularly in the case of the QDM-CQL, a more formal evaluation of executing the checks would be necessary to ensure the syntax is executing the intended purpose of the DQ check.

## Conclusions

This study demonstrates the feasibility of a data element-function conceptual model for indexing and categorizing DQ checks and being able to extract them at scale through the use of an NLP system. The results also reveal the heterogeneity in DQ checks among two networks explored, primarily due to the intended purpose of the DQ checks. The conceptual model provides a promising direction for enabling shared representation and comparison amongst DQ checks.

## Additional File

The additional file for this article can be found as follows:

10.5334/egems.289.s1 Supplemental Material. File that contains all supplemental material mentioned.
