# Supplementary material for: A Data Element-Function Conceptual Model for Data Quality Checks
Source: EGEMS (Wash DC). 2019 Apr 23;7(1):17. doi: 10.5334/egems.289 (PMC6484368; doi:10.5334/egems.289)

List of Supplemental Text, Tables and Figures

- Text: Results of additional OHDSI DQ checks
- Table S1: Allowable terms for domain descriptions of additional OHDSI DQ check constructs
- Figure S1: Horizontal bar charts of the percentage of DQ checks specific to OHDSI represented by corresponding DQ harmonization categories
- Figure S2: Horizontal bar charts of the percentage of DQ checks specific to CESR represented by corresponding DQ harmonization categories
- Figure S3: Heat map of the additional DQ check domains specific to OHDSI

Text: Results of additional OHDSI DQ checks

As a supplemental comparison, the additional 67 DQ checks for OHDSI were manually mapped to suggested domains (**Supplemental Material, Table S1**) and are summarized in a heat map (**Supplemental Material, Figure S3**). These DQ checks are generally more targeted than the other 172 OHDSI DQ checks by providing more actionable evaluations (e.g., meeting a set count or threshold). The most common data element domains were Internal ID (15; 22 percent of the DQ checks), Persons (13; 19 percent), Medication (6; 9 percent), Age (5; 7 percent), and Records (5; 7 percent). The most common function domains were Count (18; 27 percent), Invalid (17; 25 percent), Threshold (14; 21 percent), and Percentage (10; 15 percent). The most common pairings between data element domains and function domains were Internal ID-Count (9; 13 percent), Persons-Percentage (7; 10 percent), and Internal ID-Invalid (4; 6 percent).

Table S1: Allowable terms for domain descriptions of additional OHDSI data quality check constructs*

Domain	Domain Definition	Count of Checks in Domain
Data Elements (DEs)		
Age	DEs related to age-specific variables (e.g., specific to already calculated age values)	5
Birth	DEs related to birth-specific variables (e.g., specific to date-oriented values)	2
Condition	DEs related to condition-specific variables	3
Death	DEs related to death-specific variables	3
Ethnicity	DEs related to ethnicity-specific variables	1
Insurance	DEs related to insurance-specific variables	1
Internal ID	DEs defined by internally utilized constructions	15
Lab	DEs related to lab-specific variables	1
Medication	DEs related to medication-specific variables	6
Monthly Trend	DEs that are the examination of a change when comparing months	2
Persons	DEs that examines only persons (i.e., patients)	13
Procedure	DEs related to procedure-specific variables	1
Provider	DEs related to provider-specific variables	3
Race	DEs related to race-specific variables	1
Records	DEs that refer to rows in a particular table (not necessarily specified, such as persons or visits)	5
Visit	DEs that examines visit-record related variables	3
Vital	DEs related to vital-specific variables (e.g., weight)	2
Functions		
Count	Measures the count of DE relative to a certain specification (e.g., count of distinct concepts)	18
Density	Examines the concentration of a particular DE within a target DE (e.g., number of patients per provider specialty)	2
Invalid	Examines if a particular specification is appropriate for a target DE (e.g., concept from a specific vocabulary)	17
Mapping	Examines if a DE has an appropriate correspondence for a particular specification	1
Missing	Measures the availability of a DE given some specification	1
Percentage	Measures the percentage of a DE given some specification	10
Ratio	Measures whether or not a target DE relative to another DE fits a specific comparison (e.g., number of patients to number of provider)	4
Threshold	Measures DE relative to an imposed cutoff (e.g., age < 0)	14

*Because these data quality checks were examined as supplemental material and did not have the NLP methodology applied to them, there is no column listing possible terms or a count of unique terms.

Figure S1: Horizontal bar charts of the percentage of DQ checks specific to OHDSI represented by corresponding DQ harmonization categories

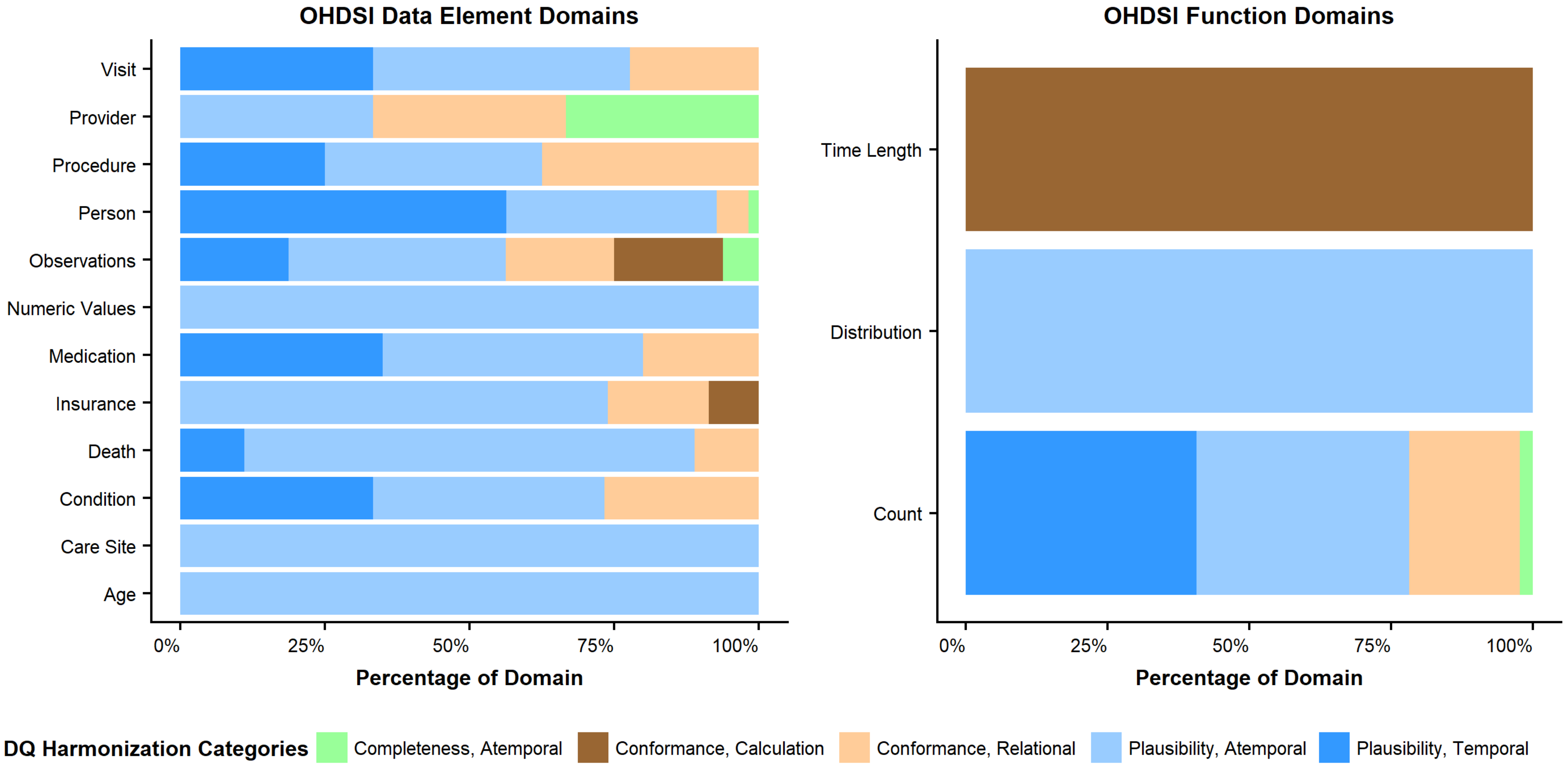

Figure S2: Horizontal bar charts of the percentage of DQ checks specific to CESR represented by corresponding DQ harmonization categories

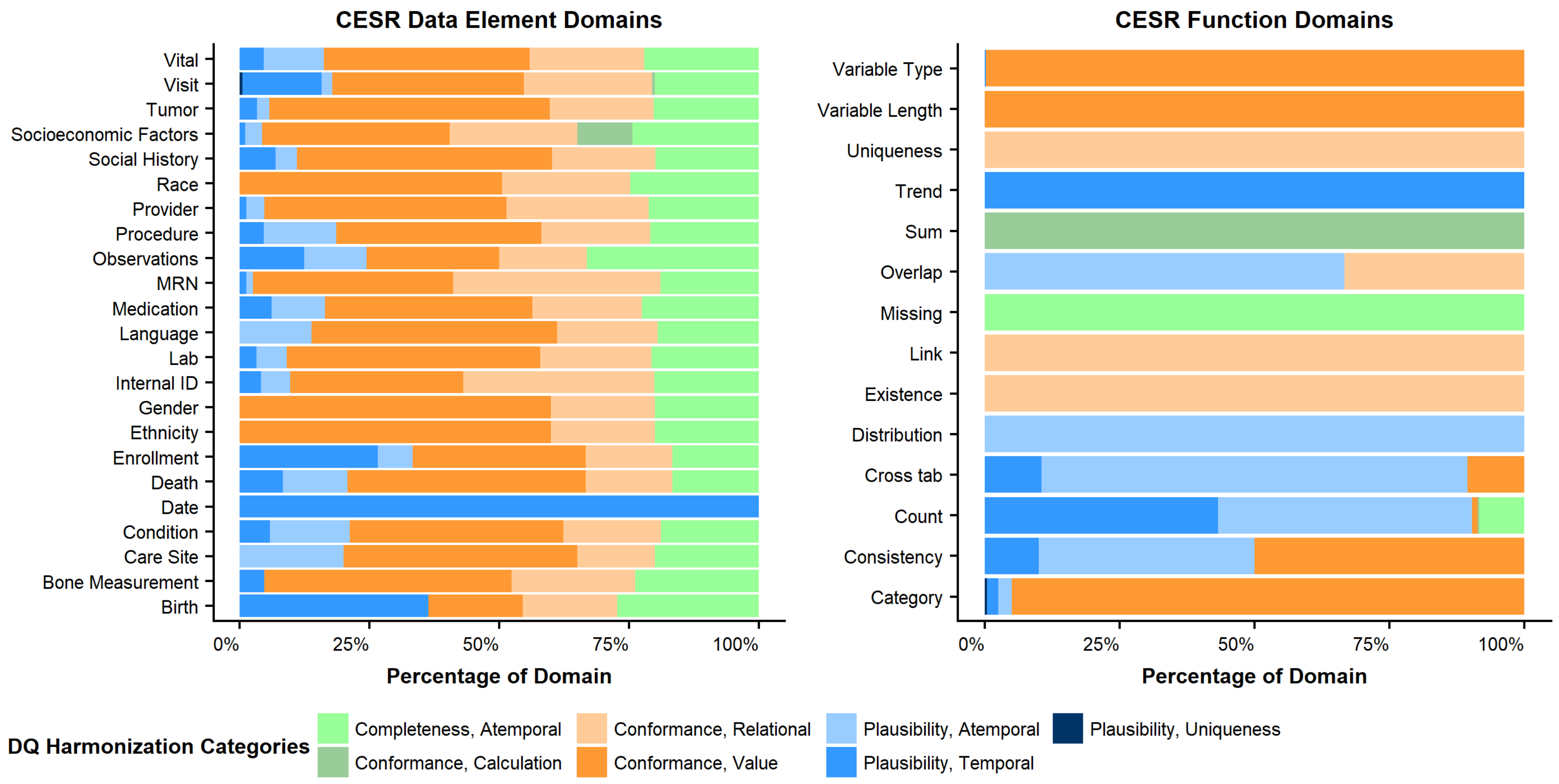

Figure S3: Heat map of the additional DQ check domains specific to OHDSI

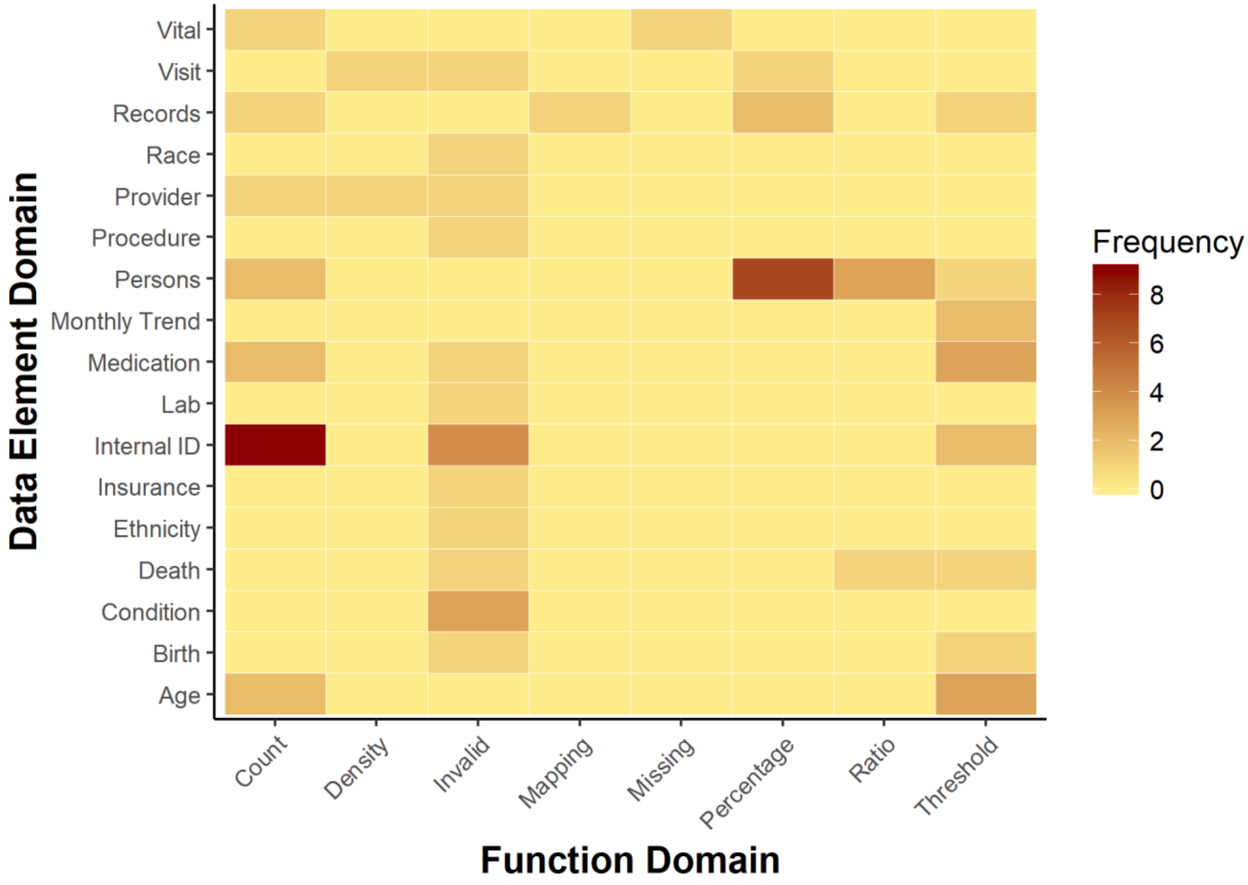

Supplement: Supplemental Material. — File that contains all supplemental material mentioned. [file egems-7-1-289-s1.pdf]
